# Timeout? The Epidemiology of Pediatric Sports Injuries During the COVID-19 Pandemic

**DOI:** 10.5435/JAAOSGlobal-D-21-00092

**Published:** 2022-04-08

**Authors:** Jacob T. Wild, Yash V. Kamani, John M. Bryan, Taylor N. Hartman, Lauren M. Spirov, Neeraj M. Patel

**Affiliations:** From the Ann & Robert H. Lurie Children's Hospital of Chicago, Chicago, IL.

## Abstract

**Background:**

The COVID-19 pandemic resulted in closure of schools and playgrounds while requiring social distancing, changes that likely affected youth sports participation. The purpose of this study was to identify changes in the epidemiology of pediatric sports injuries during the COVID-19 pandemic.

**Methods:**

This retrospective cohort study included patients between the ages of 4 and 18 years who presented to orthopaedic clinics within a single children's hospital network with an acute injury sustained during athletic activity between March 20, 2020, and June 3, 2020 (the strictest period of state-level shelter-in-place orders). These patients were compared with those within the same dates in 2018 and 2019. Chi square and Mann-Whitney *U* tests were used, as appropriate.

**Results:**

Significantly less sports injuries were seen during the pandemic (n = 257) compared with the same dates in 2018 (n = 483) and 2019 (n = 444) despite more providers available in 2020 (*P* < 0.001). During the pandemic, patients with sports injuries were younger (median age 11 versus 13 years, *P* < 0.001) and had less delay in presentation (median 5 versus 11 days, *P* < 0.001). A higher proportion were White (66.9% versus 47.7%, *P* < 0.001), privately insured (63.4% versus 48.3%, *P* < 0.001), and seen at a nonurban location (63.4% versus 50.2%, *P* < 0.001). Most sports injuries during the pandemic were fractures (83.7%). Although 71.4% of all injuries in the prepandemic period occurred in the context of formal sports, only 15.2% were sustained in a formal athletic context in 2020 (*P* < 0.001). The frequency of surgical treatment was higher during the pandemic (14.8% versus 7.8%, *P* = 0.001), mainly because most of these injuries were fractures requiring surgical intervention.

**Conclusions:**

Fewer sports injuries were seen in the outpatient setting during the COVID-19 pandemic, and most of these injuries were fractures and occurred outside of organized sports settings. Patients were more likely to be White, privately insured, and seen at a nonurban location.

The COVID-19 pandemic was officially declared by the World Health Organization on March 11, 2020.^1^ The high morbidity and mortality of this virus have resulted in extensive public health efforts to minimize the pandemic's ability to severely overwhelm health systems. Social distancing, widespread school and playground closures, and sport cancellations were some of the most commonly implemented initiatives because such measures have been effective in the past.^2,3^ These policies dramatically changed the daily lives of children and their participation in sports and group activities. Such shifts in activity may alter the epidemiology of sports-related musculoskeletal injuries in the pediatric population.

Researchers have started to assess the effect of the COVID-19 pandemic on musculoskeletal disease incidence and treatment. Notable findings from previous studies include a decrease in fracture incidence, an increase in fractures occurring at home, modifications in standard treatment patterns, and a decrease in outpatient visits compared with previous years.^4,5^ The dynamics between the COVID-19 pandemic and musculoskeletal injury incidence and treatment are likely complex and warrant additional characterization. This is particularly salient for pediatric sports injuries, which may be affected substantially by school closings and other factors.

Understanding epidemiologic trends can help identify areas of additional investigation and ultimately lead to improved injury prevention, access to care, resource utilization, and clinical outcomes. The goal of this study was to examine the epidemiology of acute pediatric sports injuries during the COVID-19 pandemic compared with previous years. The central questions of this investigation were as follows: (1) Was there a decrease in the overall volume of acute pediatric sports injuries during the COVID-19 pandemic? (2) Were there differences in injury context, mechanism, and type during the pandemic compared with previous years? (3) Was the demographic profile of children with sports injuries different during the pandemic compared with previous years?

## Methods

Institutional review board approval was obtained for this retrospective cohort study. Patients were included if they were between the ages of 4 and 18 years and presented to outpatient orthopaedic surgery and sports medicine clinics at a large, tertiary children's hospital network with an acute sports or sports-type injury between March 20 and June 3 of 2018, 2019, or 2020. The strictest state-level shelter-in-place guidelines were effective between March 20, 2020, and June 3, 2020, and included closure of public schools, beaches, parks, running and cycling paths, and recreational facilities. In addition, social distancing of six feet was recommended, therefore prohibiting all close-contact group sports.^6^ To ensure comprehensive and accurate patient inclusion and exclusion, every outpatient orthopaedic visit at our institution during these dates was screened individually rather than querying solely based on diagnosis or procedural coding.

A “sports injury” was defined as an acute injury sustained during a formal or informal sporting or athletic activity (ie, individual or team sports and fitness activities, such as running, cycling, and exercising). A “sports-type” injury was defined as a notable, often surgical, injury that is commonly sustained during sports activities but may also occur in a nonsports environment (ie, a fall in the home and during unstructured play at a playground). These included anterior cruciate ligament (ACL) and other knee ligament injuries, patellar and shoulder dislocations, meniscus injuries, and concussions regardless of the context of injury. Participants were excluded if they presented for chronic pain, chronic injuries without acute traumatic exacerbation, or a nonsports or sports-type injury. Medical record review was extended until June 20 of each year (for 2018, 2019, and 2020) to capture individuals who may have been injured during the study period but whose initial presentation was not until a later date. Patients from 2020 were then compared with those who were injured within the same dates (March 20 to June 3) in 2018 and 2019.

Demographic, administrative, and clinical elements were collected from the medical record, including age, sex, race, insurance type, provider, clinic location, provider availability, extremity, limb/joint affected, site of injury, diagnosis, injury classification, dates of injury, presentation, mechanism of injury, place/context of injury, activity during injury, and type of treatment. In 2020, our division comprised 13 pediatric orthopaedic surgeons (two specializing in sports medicine), four nonsurgical sports medicine pediatricians, and 13 independent nurse practitioners. Information regarding provider time away was tabulated for each year to take into account any differences in annual provider availability. Of note, one sports surgeon was not yet at the institution in the 2018 study period, and one general pediatric orthopaedic surgeon and one nonsurgical sports pediatrician were not yet present in 2018 and 2019. Accordingly, those providers were counted as “unavailable” for each day of the applicable study periods. Although telemedicine became a much more common medium later in the pandemic, it was in its infancy at our institution during the study period and was rarely used for acute injuries. Weather information, including daily precipitation rates and temperatures, was obtained from the National Oceanic and Atmospheric Administration publicly available data.

Statistical analysis was completed with SPSS for Macintosh, version 24.0 (IMB). Standard descriptive statistics were calculated for demographic variables. Specifically, medians were reported with interquartile range. The Kolmogorov-Smirnov test was used to test for normality of continuous variables. Independent samples Student *t*-tests were used for comparison of means while the Mann-Whitney U test was used for comparison of nonparametric continuous variables. Categorical variables were analyzed using chi square or Fisher exact tests, as appropriate. A significance threshold of *P* < 0.05 was applied for all statistical tests.

## Results

Significantly less sports injuries were seen during the pandemic (257 in 76 days) compared with those on the same dates in 2018 (n = 483) and 2019 (n = 444) despite more providers available in 2020 (*P* < 0.001). Statistical details are summarized in Table 1. During the pandemic, patients with sports injuries were younger and had less delay in presentation. A markedly higher proportion of these patients were White, privately insured, and seen at a nonurban clinic location compared with the prepandemic cohort.

**Table 1:** Demographic Comparison of Sports Injuries During and Before the COVID-19 Pandemic^a^

	Pandemic	Prepandemic	*P*
Age, y	11 (4)	13 (4)	**<0.001**
BMI	20 (6)	21 (6)	**0.01**
Sex			0.07
Female	117 (45.5)	363 (39.2)	
Male	140 (54.5)	564 (60.8)	
Race			**<0.001**
White	172 (66.9)	442 (47.7)	
Latinx	26 (10.1)	154 (16.6)	
Black	9 (3.5)	122 (13.2)	
Asian	13 (5.1)	32 (3.5)	
Others	37 (14.4)	177 (19.1)	
Insurance			**<0.001**
Private	163 (63.4)	448 (48.3)	
Public	94 (36.6)	467 (50.4)	
Others	0 (0)	12 (1.3)	
Days to presentation	5 (4)	11 (8)	**<0.001**
Clinic location			**<0.001**
Urban	94 (36.6)	462 (49.8)	
Nonurban	163 (63.4)	465 (50.2)	
Type of provider			**0.003**
Sports specialist	24 (9.3)	157 (16.9)	
Other provider	233 (90.7)	770 (83.1)	

a Values reported as median (interquartile range) for age, body mass index, and days to presentation; n (%) for all others. Significance was set at p<0.05 for this study.

In 2020, a greater percentage of acute sports injuries were fractures and involved the upper extremity (Table 2). There were less injuries related to collisions or noncontact running and jumping mechanisms. Furthermore, while 71.4% of injuries in the prepandemic years occurred in a formal sporting context, this only made up 15.2% of the patients seen in 2020. Differences were observed in the type of athletic participation during the time of injury and the type of primary treatment rendered. A higher proportion of patients were treated surgically in 2020, most of which were fractures (Table 2). Overall, no notable difference was observed in type of sports participation, resulting in a surgical versus nonsurgical injury. When analyzing fractures specifically, 34 of 217 sports-related fractures (15.7%) required surgery during the pandemic compared with 32 of 511 (6.3%) in previous years (*P* < 0.001). “Sports-type” injuries (ie, ACL tears, meniscus injuries and dislocations, as defined in the previous section) accounted for 15.7% of the prepandemic injuries, but only 2.3% in 2020 (*P* < 0.001). Specifically, less concussions, patellar and shoulder dislocations, knee ligament injuries, and meniscus tears were seen during the COVID-19 pandemic (Table 3).

**Table 2:** Clinical Comparison of Sports Injuries During and Before the COVID-19 Pandemic^a^

	Pandemic	Prepandemic	*P*
Extremity			**<0.001**
Upper	195 (75.9)	478 (51.6)	
Lower	57 (22.2)	400 (43.1)	
Head/spine	5 (1.9)	49 (5.3)	
Type of injury			**<0.001**
Fracture	215 (83.7)	511 (55.1)	
Sprain/strain	6 (2.3)	165 (17.8)	
Contusion	6 (2.3)	70 (7.6)	
Dislocation	3 (1.2)	45 (4.9)	
Concussion	3 (1.2)	57 (6.1)	
Rupture/full-thickness tear	2 (0.8)	41 (4.4)	
Others	22 (8.6)	38 (4.1)	
Mechanism of injury			**<0.001**
Fall from height	199 (77.4)	351 (37.9)	
Collision	23 (8.9)	306 (33.0)	
Noncontact running/jumping	18 (7.0)	179 (19.3)	
Others	17 (6.6)	91 (9.8)	
Setting of injury			**<0.001**
Home	100 (38.9)	81 (8.7)	
Sports	39 (15.2)	662 (71.4)	
School	0 (0)	71 (7.7)	
Others	118 (45.9)	113 (12.2)	
Activity at the time of injury			**<0.001**
Bicycling	135 (52.5)	102 (11.0)	
Skating/rollerblading	21 (8.2)	29 (3.1)	
Basketball	16 (6.2)	125 (13.5)	
Gymnastics	15 (5.8)	37 (4.0)	
Soccer	12 (4.7)	210 (22.7)	
Baseball/softball	1 (0.4)	87 (9.4)	
Running	8 (3.1)	49 (5.3)	
Football	6 (2.3)	33 (3.6)	
Others	43 (16.7)	255 (27.5)	
Primary treatment			**<0.001**
Removable brace/splint	106 (41.2)	429 (46.3)	
Cast	97 (37.7)	200 (21.6)	
Surgery	38 (14.8)	72 (7.8)	
Observation	11 (4.3)	140 (15.1)	
Physical therapy	5 (1.9)	80 (8.6)	
Others	0 (0.0)	6 (0.6)	

a Values reported as n (%). Significance was set at p<0.05 for this study.

**Table 3:** Comparison of “Sports-type” Injuries During and Before the COVID-19 Pandemic^a^

	Pandemic	Prepandemic
“Sports-type” injury	6 (2.3)	146 (15.7)
Concussion	3 (1.2)	57 (6.1)
Patellar dislocation	1 (0.4)	31 (3.3)
Anterior cruciate ligament tear	1 (0.4)	28 (3.0)
Other knee ligament injury	0 (0)	16 (1.7)
Meniscus tear	1 (0.4)	9 (1.0)
Shoulder dislocation	0 (0)	5 (0.5)
Other injuries	251 (97.7)	781 (84.2)

a Values reported as n (%).

Given the additional clinicians added to our division between 2018 and 2020, less providers were available in the prepandemic period. In the 2018 and 2019 study periods (152 total days), there were 598 provider-unavailable days compared with 142 provider-unavailable days in the 76-day study period in 2020 (*P* < 0.001). The same trend was found for the six sports specialists in the department (217 unavailable days in the prepandemic years versus 28 in 2020; *P* < 0.001). No difference was observed in the mean temperature or proportion of rainy days in 2020 compared with those in the prepandemic periods.

## Discussion

The COVID-19 pandemic and subsequent government guidelines led to an altered sports environment for children. With schools closing and organized sports curtailed because of social distancing, the landscape in 2020 for pediatric sports injuries was different than in the past years. The epidemiological effect of this dynamic has begun to be researched.^7^ However, to our knowledge, this study was the first to identify changes in the volume and demographics of pediatric sports injuries during the COVID-19 pandemic. In addition to less total injuries during the pandemic, we found that a markedly greater percentage of patients were White, privately insured, and seen at nonurban clinics. A higher proportion of sports injuries were fractures and occurred outside of a formal sports setting. Understanding these epidemiologic trends may have implications on access to care and resource utilization while providing opportunities to develop at-home injury prevention programs.

A number of recent publications have studied the effect of the COVID-19 pandemic on orthopaedic practice, especially in the adult population.^5,8-10^ One recent study concluded that markedly less pediatric fractures were seen during the pandemic compared with the past years. The authors noted a decrease in playground injuries and an increase in fractures occurring at home. The reduction in fracture incidence was steepest for sports-playing older children (nearly five-fold).^4^ Although the study by Bram et al focused on fractures sustained by a variety of mechanisms, our study on sports injuries similarly found that less patients were seen in 2020 compared with previous years. A markedly greater proportion of injuries occurred at home during the pandemic, with fewer seen in a formal sports context and at school. We also found that a greater percentage of injuries during the pandemic were due to a fall from height (ie, while cycling or during other athletic activities) and fewer from collisions and noncontact running or jumping. Finally, the proportion of fractures increased in 2020, whereas the rate of lower extremity injuries decreased. These findings reflect a clear shift in the patterns of sports injuries seen in the pediatric population after the COVID-19 shelter-in-place restrictions. As the type and context of sports injuries evolve, improved guidelines for injury prevention are necessary. Some institutions have started warning about at-home injuries during the pandemic.^11,12^ Recommendations from national organizations are more focused on safe return-to-play guidelines.^13,14^

The median patient age was lower during the pandemic by 2 years. This may be due, in part, to less high school–aged children playing organized sports in 2020. Previous studies have shown that more than half of high school students in the United States participate in organized athletics.^15^ With most of these adolescents no longer playing sports, this likely resulted in less overall injuries, a younger median age, and a difference in the types of injuries seen. For example, 1.9 million children sustain a sports-related concussion each year, with adolescent teens at highest risk.^16^ The incidence of ACL tears in the pediatric population is also far higher between the ages of 15 and 18 years than in younger children.^15^ In this study, there was a stark decrease in the number of “sports-type” injuries seen (ie, concussions, ACL tears, and others), resulting in a higher proportion of injuries that were fractures in younger children. It is likely that the halt of organized high school sports contributed to these changes.

Differences in the time to clinic presentation were seen during the COVID-19 pandemic. Interestingly, although previous studies in other specialties have noted delays in presentation^17,18,19,20^ and the study by Bram et al^4^ noted no change, patients in this study experienced a shorter wait in 2020 than in previous years (5 versus 11 days). This may be due to a greater proportion of injuries being fractures during the pandemic. At our institution, a concerted effort is made to schedule initial clinic evaluation for known fractures between 3 and 5 days after injury. In the absence of the typical number of other sports injuries that may not require as urgent attention (ie, sprains, strains, contusions, and others), the median time between injury and presentation was reduced during the shelter-in-place period of 2020. In addition, it is possible that some parents and guardians were more likely to be at home during the pandemic and therefore had greater flexibility to attend medical appointments.

Such changes also likely affected the type of treatment that was rendered. Specifically, 14.8% of the patients underwent surgery in 2020 compared with 7.8% in the prepandemic years. Similar to the time to presentation data, this was likely related to an increased proportion of injuries being fractures requiring surgical intervention while diagnoses such as ACL and meniscus tears were far less common. Of note, delays of elective surgeries likely did not affect this study because the focus was on date of initial outpatient evaluation rather than timing of surgery. Interestingly, although Bram et al^4^ reported no change in the percentage of fractures requiring surgery, our study found that 15.7% of the sports-related fractures required surgery during the pandemic compared with 6.3% in previous years. This may suggest that while the overall number of sports injuries was lower in 2020, fractures sustained during this period were of greater severity than earlier. In addition, a possibility is that unconscious surgeon bias during the pandemic could have resulted in a lower threshold to operate, although our institutional quality initiatives, indications, conferences, and other programs aim to maintain consistency in surgical indications. Such data again point to the importance of developing prevention guidelines for at-home injuries.

Several demographic changes were seen during the COVID-19 pandemic. In 2020, patients seen at our clinics for an acute sports injury were more likely to be White and privately insured than in 2018 and 2019. In addition, these children were seen more frequently in nonurban locations. The underlying reasons for these findings are beyond the scope of the data, but some potential factors could have contributed. One possible explanation could be differences in the sports played by children from different communities, resulting in varying injury risk profiles. Another is that wealthier communities or those in nonurban areas may have more open space and better access to resources (ie, parks, fields, and club or traveling teams that compete outside of the state) needed to participate in sports and athletic activities than those living in urban areas. A state-wide survey in Tennessee found that 16% of Black parents thought it was safe for children to participate in sports during the pandemic compared with 44% of White parents. Furthermore, 51% of the respondents with a household income of >$75,000 agreed that youth sports participation is safe versus 25% of those with an annual income of <$25,000.^21^ Such trends may have played a role in this study as well. In addition, those living in urban areas may not have access to safe, affordable, and reliable transportation during a pandemic, making it more difficult to participate in recreational activities or receive care. Therefore, while patients were able to obtain a clinic appointment more quickly during the COVID-19 pandemic, this improvement was more likely to be experienced by White, privately insured patients in nonurban areas.

Previous research has suggested that the COVID-19 disproportionately affects Black and Hispanic communities with higher mortality and infection rates than White communities.^22^ Minority populations were also more likely to be essential workers^22^ and live in close communities,^23^ making social distancing a bigger challenge. Similar factors may have led to less athletic participation or seeking of health care for minor injuries in these populations during the pandemic. Furthermore, there are known disparities in pediatric orthopaedic and sports medicine care along the lines of race and insurance status. These have been well described in the management of ACL injuries and fractures.^24,25,26,27,28^ Cultural differences in seeking care and trust in the medical establishment, which have already been shown to vary between racial groups based on historic and present experiences,^29,30,31,32,33,34^ may also have factored into families' decisions to seek care for less severe sports injuries during the pandemic. Given these known disparities regarding both the COVID-19 and pediatric orthopaedic care, it is possible that similar issues played a role in the results of this study. Ultimately, the exact reasons for these differences are beyond the scope of our data. Additional research should focus on the demographic shifts in pediatric sports injuries during the pandemic and investigate potential barriers to care.

This study is limited by its retrospective nature and its inclusion of a single tertiary referral center. Although it is possible that referral patterns to our institution may have changed between 2018 and 2020, internally available business data suggest that the referral volume has only increased within those years. The findings of this study may not be generalizable to other regions because of differing regulations surrounding COVID-19. In addition, variation in sports played, demographics, and social and medical infrastructure are all factors that may affect these findings in other areas. In addition, a possibility is that some children did not seek care during the pandemic or sought care elsewhere rather than at our center, especially for minor injuries. Although our institution is the largest provider of pediatric orthopaedic care in the region, it is possible that such phenomena may have affected the findings of this study.

In conclusion, a number of epidemiologic changes were noted in pediatric sports injuries during the COVID-19 pandemic. Overall, the number of these injuries was lower and more were sustained at home. A higher proportion of injuries consisted of fractures. During the pandemic, pediatric patients with acute sports injuries were younger and more likely to be White, privately insured, and seen at nonurban clinics. These findings provide opportunities for at-home injury prevention guidance and additional research regarding barriers to care during a pandemic.
